# Clinical and Genetic Review of Hereditary Acral Reticulate Pigmentary Disorders

**DOI:** 10.1155/2017/3518568

**Published:** 2017-10-23

**Authors:** H. Alshaikh, F. Alsaif, S. Aldukhi

**Affiliations:** Department of Dermatology, College of Medicine, King Saud University, Riyadh, Saudi Arabia

## Abstract

Reticulated pigmentation is a unique pigmentary change caused by a heterogeneous group of hereditary and acquired disorders. This pigmentation is characterized by a mottled appearance, with lesions that vary in size and pigmentary content. This review discusses the hereditary group of the reticulated pigmentation disorders, such as dyschromatosis symmetrica hereditaria, dyschromatosis universalis hereditaria, and reticulate acropigmentation of Kitamura. The clinical presentation and histopathological features often overlap, making diagnosis difficult. However, each of these hereditary conditions possesses a unique genetic mutation, and genetic analysis is thus more useful in the diagnosis of these conditions. This article delivers an update regarding the clinical features, detailed histopathological description, and genetic information concerning hereditary reticulate pigmentary disorders and aims to provide useful background for use by clinical dermatologists and histopathologists when approaching this group of hereditary disorders.

## 1. Introduction

Pigmentary disorders encompass a broad variety of hypo- and hyperpigmentation disorders. They can be hereditary or acquired and have many causes, including abnormalities in melanoblast migration, melanosome development or transfer, changes in the numbers of melanocytes, or defects in melanin synthesis [1, 2]. The clinical manifestations of pigmentary disorders vary; they can present as circumscribed, diffuse, linear, or reticular pigmentary changes. Circumscribed patterns are often seen in postinflammatory hyperpigmentation and melasma. Diffuse pigmentation can be noted in familial diffuse melanosis and Addison's disease. Incontinentia pigmenti, linear and whorled nevoid hypermelanosis, and progressive cribriform and zosteriform hyperpigmentation follow a linear pattern. Reticulate pigmentation, which is characterized by a mottled appearance and variation in the size and pigmentary content of lesions [3], is seen in reticulate acropigmentation of Kitamura, confluent and reticulated papillomatosis of Gougerot, and Carteaud and other rare dermatoses.

This review focuses on hereditary pigmentary disorders presenting with the reticulated pattern, whether hypo- or hyperpigmented, that follow an acral distribution. With advancements in genetic analysis and molecular techniques, identification of the causative mutations and their respective genes has improved our understanding and provided a useful tool for enhancing accuracy in diagnosing these disorders. The clinical manifestations, genetic backgrounds, and histopathological features of these disorders are compared and summarized in Tables 1, 2, 3, and 4. This information, along with a discussion of treatment options reported in the literature, is organized in such a way as to aid in the differentiation and diagnoses of these uncommon disorders.

## 2. Dyschromatosis Symmetrica Hereditaria

Dyschromatosis symmetrica hereditaria (DSH), also known as acropigmentation of Dohi, was first described by Toyama in 1910 as an unknown hyperpigmentation disorder of the distal extremities [4]. In 1929, Toyama named the disease DSH [5], and several cases have since been reported from various parts of the world, including India, Europe, and South America. Most of these cases have been reported in Japan and China [6–10].

**Figure 1:**
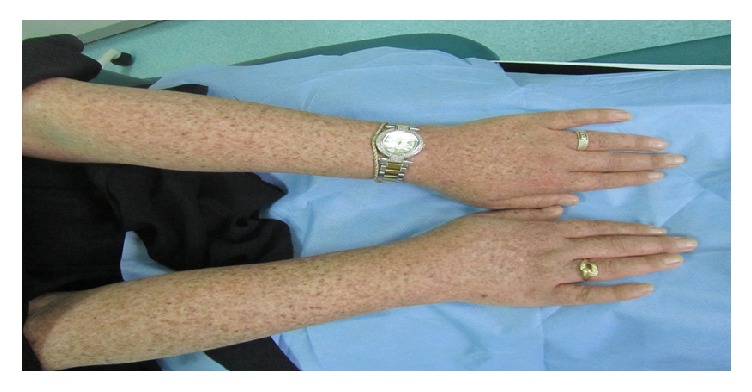
Dyschromatosis symmetrica hereditaria: extensive mixed hypo- and hyperpigmented macules over the dorsal aspect of the upper limb.

The course of DSH starts early in life or in infancy, with progression halting around adolescence but remaining with the patient for life without showing significant changes in color or distribution [11, 12]. Skin lesions are characterized by a mixture of hypo- and hyperpigmented macules of various sizes arranged in a reticulated pattern on the dorsal aspects of the extremities (Figure 1), and small freckle-like pigmented macules on the face with no areas of hypopigmentation have been noted in some patients [13, 14]. The palms, soles, and mucosa are usually spared. The skin lesions do not exhibit photosensitivity but are more pronounced after sun exposure [15, 16].

A unique pattern of hair involvement can also be seen, with hair hyperpigmentation in hypopigmented macules and hair hypopigmentation in hyperpigmented or normal skin [17].

Dermoscopy of the pigmented areas can show reticulated hyperpigmented spots, reticulate pigmented spots, monotonous pigmented spots, reticulated hypopigmented spots, or monotonous hypopigmented spots [18].

While usually arising as an isolated disorder, associated conditions or complications sometimes seen include acral hypertrophy, depression, psoriasis [19], dental anomalies [17], aortic valve sclerosis [20], and neurological manifestations in the form of brain calcification, mental deterioration, dystonia [20, 21], and intracranial hemangioma [22].

Histological sections of hyperpigmented macules show increased melanin pigment in the basal layer along with pigmentary incontinence and decreased melanin pigment in hypopigmented macules [23–25]. Increased melanocyte size and dendrite elongation, which indicate active transfer of melanosomes to keratinocytes, are also seen in the hyperpigmented macules. Melanocytes are fewer in number in the hypopigmented areas and in the surrounding normal skin [24].

Hyperpigmented lesions examined using electron microscopy occasionally reveal many small melanosomes in keratinocytes and a few small melanosomes in melanocytes. In hypopigmented lesions, small immature melanosomes in the melanocytes are seen along with cytoplasmic vacuolization, mitochondrial degeneration, and irregularly shaped nuclei, indicating apoptotic changes [24, 25]. Additionally, a cell-to-cell contact phenomenon between Langerhans cells and lymphocytes has been observed in the hypopigmented lesions, which is the same phenomenon seen in active vitiligo lesions [26]. Although sporadically reported [27], DSH is recognized as a familial disease with an autosomal dominant pattern of inheritance with high penetrance [9]. It is caused by adenosine deaminase acting on an RNA1 gene mutation (ADAR1) located on chromosome 1q21.3, which encodes an RNA editing enzyme [9, 28]. More than 100 mutations have been described in the ADAR1 gene, and most are missense in origin [13]. Carriers of the ADAR1 mutation show variable expressivity, attributable to environmental factors such as ultraviolet light exposure or early-life infections [13].

Recently, associations between DSH and Aicardi-Goutières Syndrome (AGS) have been shown. A heterozygous mutation of ADAR1 encoding p.Gly1007Arg leads to skin and neurological manifestations. However, a homozygous mutation of ADAR1 p.Gly1007Arg results in AGS. In contrast, a person carrying a heterozygous mutation of ADAR1 other than p.Gly1007Arg usually has skin manifestations only [29].

Treatment is primarily for cosmetic purposes, but an effective treatment has not yet been documented [6]. One patient was treated with fractional CO_2_ laser and results were positive after 4 treatment sessions; however, further treatment trials are needed to establish the efficacy of this method [30]. Miniature punch grafting combined with a 308 nm excimer laser light is another experimental treatment that has been reported to be successful [31].

## 3. Dyschromatosis Universalis Hereditaria

Dyschromatosis universalis hereditaria (DUH) is a rare genodermatosis first reported by Toyama in 1929 and subsequently by Ichikawa and Hiraga in 1933 [32]. It was first thought to be limited to Japanese populations. However, several cases have been reported in Europe, China, Saudi Arabia, Tunisia, and India [33].

**Figure 2:**
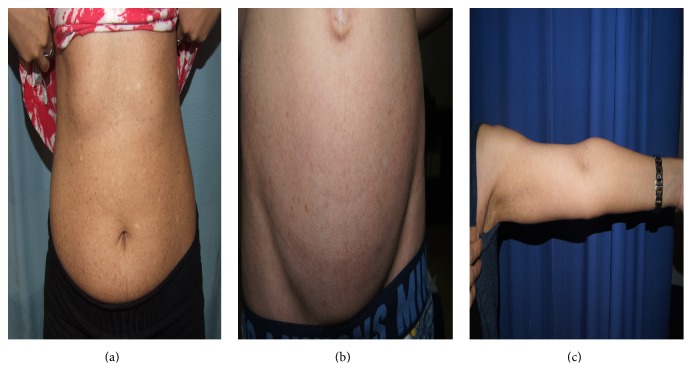
Clinical findings of dyschromatosis universalis hereditaria in a mother and her son: multiple hyper- and hypopigmented macules of varying sizes can be noted over the trunk and upper limb ((a)–(c)).

Clinically, DUH presents with reticular hyper- and hypopigmented macules of varying sizes that are generally distributed [34] (Figure 2). Moreover, the size of the macular pigmentation varies from a few millimeters to several centimeters [35]. Half of patients present with facial involvement, and the palms and soles are considered to be unusual sites of presentation. A few reports have described involvement of the oral mucosa, hair, and nails [34, 36]. In addition, DUH lesions show no atrophy or telangiectasia [37] and neither progression nor spontaneous regression with age [37, 38].

Most DUH patients have associated conditions [39]; however, the literature reports rare associations with tuberous sclerosis, photosensitivity, neurosensory hearing defects, small stature, X-linked ocular albinism [34], bilateral glaucoma, cataracts, insulin-dependent diabetes [38, 40], primary ovarian failure, and hypothyroidism [33].

The pathogenesis of DUH is still unknown, but one hypothesis suggests that it should be considered a disorder of the melanosome synthesis rate or activity and not a disorder in the number of melanocytes [28]. While DUH is usually autosomal dominant in inheritance, a few cases have been reported with autosomal recessive or sporadic inheritance [40].

Two mapped loci were initially found to be responsible for DUH 6q 24.2-q25 in two Chinese families and 12q21-q23 in an Arab family. However, there was no specific causative gene identified [41, 42]. In 2013, it was found that a missense mutation in gene ABCB6 (ATP binding cassette subfamily B, member 6) located in chromosome 2q35 was responsible for the pathogenesis of DUH [43]. The skin contains ABC transporters, responsible for molecular transportation across cell membranes, which play roles in melanosome transport to surrounding keratinocytes [33]. Though DUH is considered to be prototypically similar to DSH, they are genetically distinct disorders. The former is caused by heterozygous mutations in the ABCB6 gene, while the latter is caused by mutations in the ADAR1 gene.

Histopathological examination using a light microscope varies depending on the location from which the skin was biopsied. If taken from hyperpigmented macules, samples will show an increase in melanin in the basal layer, pigmentary incontinence, and some melanophages in the upper dermis [38]. In contrast, hypopigmented lesions exhibit decreased melanin deposition in the basal layer [36].

Under an electron microscope, the fully melanized melanosomes in the hyperpigmented macules form melanosome complexes that are not present in hypopigmented macules [34]. Therefore, the suggested mechanism of pathogenesis is that the inherited genetic defect responsible for DUH interferes with the production and distribution of the melanosome in the epidermal melanin units and does not involve a disorder in the total number of melanocytes [40].

To date, there is no definitive treatment for DUH. Patient education and reassurance are often recommended. Experimental treatment using narrow band ultraviolet B (NBUVB) therapy shows positive results after 20 sessions [37]. Another treatment reported to be successful is using Q-Switched Alexandrite Laser to treat the hyperpigmented macules of DUH; however, longer follow-up of the result is needed [39].

## 4. Reticulate Acropigmentation of Kitamura

Reticulate acropigmentation of Kitamura (RAPK) is a rare pigmentary disorder. The first case was observed in Japan in 1943, and it was later reported in the European literature by Kitamura in 1953 [44]. Griffiths was the first to conduct a study, in 1976, reported in the English literature [45]. Since then, many cases have been reported, mainly among Asian populations [46] but also in other populations worldwide [47–50].

Usually, RAPK presents in the first or second decade of life, manifesting as hyperpigmented macules on the dorsal aspects of the hands and feet and gradually extending proximally [45]. Occasionally, the eyelid [51–53], face, abdominal skin, and skin folds are affected [46], and lingual mucosal involvement has also been reported [52].

**Figure 3:**
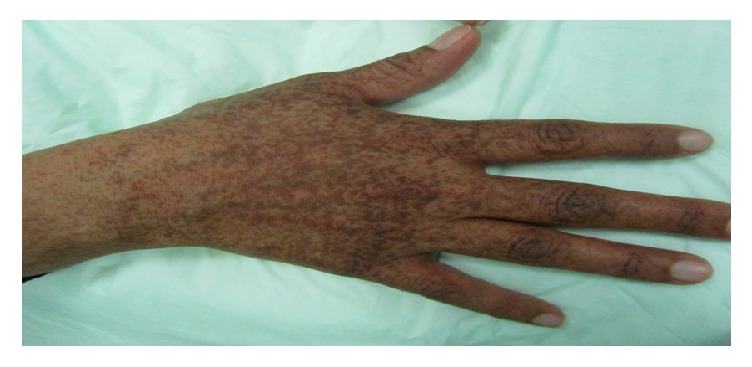
Reticulate acropigmentation of Kitamura: pronounced reticulated and confluent hyperpigmentation is noted over the dorsum of the hand.

**Figure 4:**
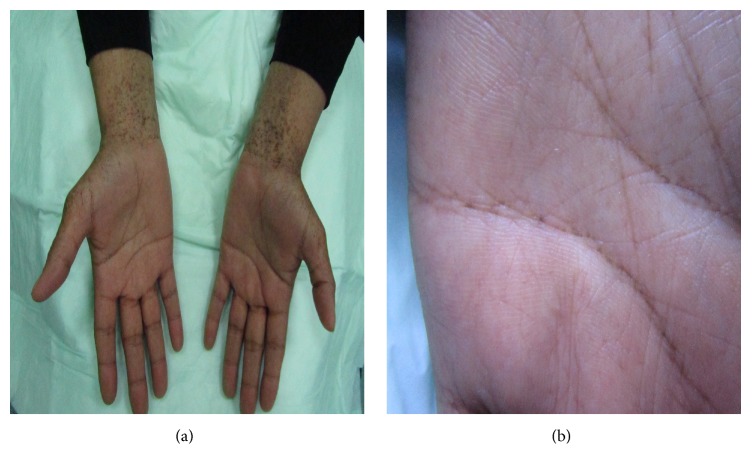
Reticulate acropigmentation of Kitamura: hyperpigmented macules over the ventral side of the distal forearm (a). Multiple pits are evident over the line of the palmar creases (b).

The typical skin morphology of RAPK includes angulated, punctate, and slightly depressed hyperpigmented macules in a reticulated pattern with no areas of hypopigmentation (Figure 3). Pits and breaks often appear in the palms (Figure 4) and soles [48, 54, 55]. A mixture of brown lines and dots forming a pigmented reticular network along with depressions is seen on dermoscopy [54, 55].

Several diseases are reportedly associated with RAPK, including plantar keratoderma, talipes equinovarus, acrokeratoelastoidosis, psoriasis, nevus spilus, nevus anemicus, acne excorie [45], nonscarring alopecia [56], and bony anomalies in the form of the absence or hypoplasia of the terminal phalanx of the toes [52].

On light microscopy, hyperpigmented lesions show thinning and elongation of the rete ridges along with hyperkeratosis without parakeratosis and increased melanin pigment in the basal layer of the epidermis without pigment incontinence [47, 55, 57]. Few inflammatory cells and melanophages can be observed in the dermis [53, 58].

Compared with the perilesional areas, examination of the hyperpigmented areas under electron microscopy shows a significant number of melanocytes in the basal layer and melanosomes in the keratinocyte cytoplasm [55, 57].

RAPK is usually inherited in an autosomal dominant pattern with high penetrance, although sporadic cases have been reported [50, 53].

In an exome sequencing study, ADAM10, a member of a disintegrin and metalloproteinase family, was found to be a causative gene for RAPK. The type of mutation in the ADAM10 gene could be a nonsense, missense, or splice site mutation [59]. Increased E-cadherin proteolysis and blister formation in patients with eczematous dermatitis were positively correlated with increased ADAM10 expression [60].

The PAX2 gene plays a vital role in ADAM10 expression, and it has been observed that increased ADAM10 expression via upregulation of PAX2 is associated with melanoma metastasis, attributed to the metalloproteinase activity of the ADAM10 gene [61]. Increased skin pigmentation in a colony of hairless mice during aging is similar to that seen in aging human skin and is associated with ADAM10 mutation, highlighting the inhibitory effect of the ADAM10 gene on melanocyte expansion [62].

The literature does not provide a consensus regarding the best therapeutic approach for RAPK. Several treatments have been tried with varied success rates. In 2014, a 532 nm Q-switched Nd:YAG laser treatment produced a significant improvement in hyperpigmented lesions, and no repigmentation was seen after 10 years of follow-up. Furthermore, no significant adverse effects were observed [58]. In another study, a Q-Switched Alexandrite Laser (755 nm) provided a positive result, resolving the pigmented lesions but allowing mild repigmentation after two years [48]. Because of increased tyrosinase activity in RAPK patients, treatment with 20% azelaic acid ointment provided positive results in a relatively short period of 2 weeks. No long-term follow-up data for this approach are available [57]. None of these treatment options are based on robust evidence; thus, a clinical trial comparing treatment modalities is needed.

## 5. Dermatopathia Pigmentosa Reticularis

Dermatopathia pigmentosa reticularis (DPR) is a rare autosomal dominant ectodermal dysplasia [63] characterized by reticular pigmentation that appears at birth or early childhood. It persists throughout life, showing no tendency toward spontaneous fading [64]. A few reported cases, most from Europe, can be found in the literature.

**Figure 5:**
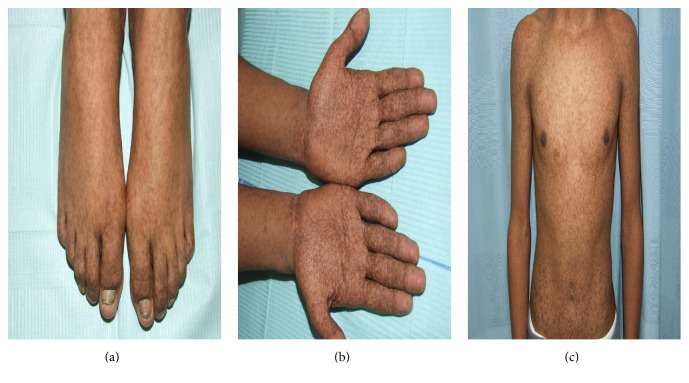
Dermatopathia pigmentosa reticularis: extensive hyperpigmentation in a reticulated pattern is noted on the dorsal aspect of the feet and the palms ((a)-(b)). Persistent reticulated hyperpigmentation that is widespread over the trunk (c).

A diagnosis of DPR is often made clinically because all reported cases have many common features and share a classical triad of universal reticulate hyperpigmentation (Figure 5), nonscarring alopecia, and onychodystrophy. In addition to this diagnostic triad, patients can present with other dermatological manifestations, such as adermatoglyphia, palmoplantar hyperkeratosis, hypohidrosis, or hyperhidrosis [63]. A number of cases have been reported with corneal involvement (e.g., Salzmann's nodular degeneration of the cornea) or punctate superficial spots in the cornea [64].

Histopathological manifestation of the reticulate pigmentation of DPR is not diagnostic. Features include mild orthokeratosis, papillomatosis, a heavily pigmented epidermis, liquefaction degeneration of the basal layer, dermal pigmentary incontinence, melanophages, interface dermatitis, and sparse, superficial perivascular inflammation [65]. Electron microscopic examination of hyperpigmented lesions show increased numbers and sizes of melanosomes in basal keratinocytes [66].

This disorder shares an autosomal dominant mutation in the KRT14 gene located on chromosome 17q11.2-q21 with Naegeli-Franceschetti-Jadassohn Syndrome (NFJS) and can be caused by either frameshift or nonsense mutations [65, 67]. Thus, the main distinction is based on clinical manifestations; lifelong persistence of the reticular pigmentation is noted in DPR, but fading after puberty is noted in NFJS [67].

There is no specific treatment for DPR except for symptomatic management of cutaneous problems, such as palmoplantar keratoderma, for which topical retinoids and keratolytics may be useful [63].

## 6. Naegeli-Franceschetti-Jadassohn Syndrome

An infrequent autosomal dominant form of ectodermal dysplasia is NFJS [68], which is characterized by reticular hyperpigmentation, hypohidrosis with heat intolerance, and the absence of dermatoglyphics [69]. Brown-gray to brown reticular pigmentation, mostly over the abdomen, neck, trunk, axillae, groin, and perioral, periorbital, and proximal extremities, is seen [68]. Pigmentation tends to appear spontaneously and is not preceded by inflammatory changes or bullous lesions [70]. Patients usually start showing pigmentation at approximately 2 years of age and spontaneous regression after puberty, with pigmentation disappearing entirely by 60 years of age. This contrasts with hypohidrosis, which remains constant even after puberty [68]. Hypohidrosis as a result of diminished sweat gland function is the most intolerable clinical manifestation because it causes discomfort and possible collapse provoked by heat, even after mild exercise [71, 72].

Patients with NFJS have a severe enamel defect, predisposing them to early total loss of their teeth [73]. To date, all reported cases of NFJS have been associated with hypoplastic dermatoglyphics, a distinctive clinical feature of the syndrome [74]. A few cases of blistering lesions on the palms and soles have been reported among NFJS patients [69]. Additional cutaneous manifestations include diffuse palmoplantar keratoderma with linear patterns of punctate keratoses, sometimes accentuated in the creases [73], congenital great toenail misalignment, onycholysis, and subungual hyperkeratosis [68, 69]. Until recently, no growth retardation has been reported, and good health and normal intelligence have universally been described [71].

Genetic studies have recently confirmed that DPR and NFJS are allelic, with a common mutation in KRT14 [68]. The clinical features of DPR, in contrast to those of NFJS, are characterized by lifelong persistence of skin hyperpigmentation, partial alopecia, and the absence of dental defects [69]. NFJS alone has persistent hypohidrosis and palmoplantar keratoderma, with spontaneous fading of the hyperpigmentation with advanced age [69].

While NFJS is considered to be a rare autosomal dominant inherited form of ectodermal dysplasia [75], the genetic mutation was recently found to be caused by heterozygous nonsense or frameshift mutations in KRT14 [70]. These mutations were found to be localized on chromosome 17q11.2–17q21 [73].

Histological examination of the hyperpigmented lesions under light microscopy reveals increased melanin pigments in the basal layer associated with pigmentary incontinence and dermal melanophages [76]. The papillary dermis, under electron microscopy, shows increased colloid (apoptotic) and amyloid bodies, sometimes around sweat glands in the reticular dermis [67].

Patients are often advised to avoid strenuous exercise, as this may lead to collapse due to hypohidrosis [69].

## 7. Epidermolysis Bullosa Simplex with Mottled Pigmentation

Epidermolysis bullosa simplex (EBS) is a group of disorders characterized by intraepidermal blister formation occurring spontaneously or after minimal trauma, typically at birth or shortly thereafter. It is generally inherited in an autosomal dominant pattern, though autosomal recessive inheritance has been seen [77].

Epidermolysis bullosa simplex with mottled pigmentation (EBS-MP) is a rare variant of EBS that was first reported by Fischer and Gedd-Dahl in 1979 [78]. It presents as bullae at birth or in early infancy that heal without scarring. It can be generalized or localized to the extremities. Hyperpigmentation eventually occurs later in infancy or in childhood, at which point skin lesions are usually described as hyperpigmented macules in a reticular pattern often associated with hypopigmented macules. Palmoplantar hyperkeratosis, skin atrophy, and nail dystrophy can also be seen [79–81].

Photosensitivity and telangiectasia have also been described in some EBS-MP patients [82], but no extracutaneous involvement or increased risk of skin malignancy has been seen [83].

Monitoring these patients is vital because, in the neonatal period, it is initially difficult to distinguish between EBS-MP and other forms of EBS, as they commonly present with bullae at birth that decrease with age. One difference is that EBS-MP is usually followed by hyperpigmentation [77].

The most common mutation responsible for EBS-MP is a missense mutation, p.pro25leu, in the KRT5 gene (proline to leucine amino acid substitution at the 25th residue of the KRT 5 gene) [77, 84]; however, a mutation in the KRT14 gene and a recently discovered EXPH5 nonsense mutation have also been reported [85–87].

Light microscopy of hyperpigmented macules shows epidermal atrophy, increased pigmentation in the basal cells, and pigmentary incontinence. Dyskeratotic cells and basal vacuolization are also seen [80].

Electron microscopy of the hyperpigmented areas shows plenty of melanosomes within the basal keratinocytes and disorganization of the keratin filaments. Keratinocyte vacuolization is also seen [82, 88].

The only reported treatment for EBS-MP is genetic counseling and treatment of blisters during initial presentation.

## 8. Amyloidosis Cutis Dyschromica

Amyloidosis refers to a group of disorders characterized by deposition of extracellular amyloid protein in various organs. Primary cutaneous amyloidosis is characterized by skin deposition of amyloid without systemic involvement. Amyloidosis cutis dyschromica (ACD), a rare variant of primary cutaneous amyloidosis first described in 1970 [89], is primarily seen in Asian populations [88].

The clinical features of ACD are mottled, reticular, hyper- and hypopigmented macules with generalized distribution [90, 91] without involvement of mucous membranes, palms and soles, or skin appendages [91, 92]. Onset is typically during childhood, and it is asymptomatic or accompanied by mild pruritus in most cases [93].

An autosomal recessive inheritance pattern is assumed in ACD [92]; however, there have been many reported sporadic cases [91, 94, 95]. No gene has yet been found to be responsible for its manifestation [93]; however, it has been suggested that hypersensitivity to UV radiation and reduced DNA repair may play roles in the pathogenesis of ACD [96].

Amyloid deposition is believed to originate from keratinocytes [91, 97]. It is postulated that repeated epidermal damage from ultraviolet light radiation can lead to amyloid deposition as a result of cytokeratin released from keratinocyte apoptosis [98].

The literature occasionally reports systemic associations with ACD, including atypical parkinsonism [99], morphea [100], interstitial pulmonary fibrosis [101], and colon cancer [91].

Light microscopy of the hyper- and hypopigmented macules shows amorphous eosinophilic material (amyloid) in the papillary dermis, irregularly elongated rete ridges, and melanin pigment incontinence in the dermis [101]. The eosinophilic material stains positive with Congo red, showing apple-green birefringence under polarized light, indicating amyloid deposits [91]. Electron microscopy shows amyloid fibrils [100].

No well-documented approach for the treatment of ACD has been found. Various treatment modalities have been tried with mixed results; photoprotection and avoidance of sun exposure is essential [97]. Acitretin is a promising drug shown to be effective against ACD in several studies [90, 91, 93, 94]. Antioxidants and topical treatments such as urea cream and tazarotene are not effective against ACD [90, 93].

## 9. Conclusion

All of the disorders discussed in this review are inherited as autosomal dominant disorders, with the exception of amyloidosis cutis dyschromica. DPR and NFJS share a common genetic mutation in the KRT 14 gene in chromosome 17. A unique histopathological finding is noted in RAPK, namely, the absence of pigmentary incontinence of the hyperpigmented lesions.

Because the diagnosis of hereditary reticulate pigmentation based on clinical features can be difficult, genetic analysis and a detailed family history play important roles in diagnosis and genetic counseling.

## Figures and Tables

**Table 1:** Characteristic clinical features of hereditary reticulate pigmentary disorders.

Disorders	Primary skin lesion	Additional features
DSH	Mixed reticular hyper- and hypopigmentation on distal extremities	Freckle-like macules on the face Palms and soles are spared

DUH	Mixed reticular hyper- and hypopigmentation on trunk and distal extremities (generalized)	Freckle-like macules on the face Palms and soles are rarely involved

RAPK	Reticular hyperpigmented macules in distal extremities	Palmoplantar pits

DPR	Reticular hyperpigmentation of distal extremities and trunk (generalized)	Nonscarring alopecia and onychodystrophy

NFJS	Reticular hyperpigmentation of distal extremities and trunk (generalized) that fades after puberty	Palmoplantar keratoderma Hypohydrosis Absence of dermatoglyphics

EBS-MP	Blisters followed by hyper- and hypopigmented macules in reticular pattern with generalized or localized (extremities) distribution	Palmoplantar hyperkeratosis and nail dystrophy

ACD	Reticular hyper- and hypopigmented macules with generalized distribution	—

DSH: dyschromatosis symmetrica hereditaria; DUH: dyschromatosis universalis hereditaria; RAPK: reticulate acropigmentation of Kitamura; DPR: dermatopathia pigmentosa reticularis; NFJS: Naegeli-Franceschetti-Jadassohn Syndrome; EBS-MP: epidermolysis bullosa simplex with mottled pigmentation; ACD: amyloidosis cutis dyschromica.

**Table 2:** Summary of genetic differences between hereditary reticulate pigmentary disorders.

Disorder	DSH	DUH	RAPK	DPR	NFJS	EBS-MP	ACD
Mode of inheritance	AD	AD	AD	AD	AD	AD	AR
Gene mutation	ADAR1	ABCB6	ADAM10	KRT14	KRT14	KRT5	Unknown
Chromosome location	1q21.3	2q35	15q21.3	17q11.2–q21	17q11.2–q21	12q13.13	Unknown

AD: autosomal dominant; AR: autosomal recessive; DSH: dyschromatosis symmetrica hereditaria; DUH: dyschromatosis universalis hereditaria; RAPK: reticulate acropigmentation of Kitamura; DPR: dermatopathia pigmentosa reticularis; NFJS: Naegeli-Franceschetti-Jadassohn Syndrome; EBS-MP: epidermolysis bullosa simplex with mottled pigmentation; ACD: amyloidosis cutis dyschromica.

**Table 3:** Histopathological features, light microscopy.

Disorder	Light Microscopy
DSH	*Hyperpigmented macules* (i) Increase in melanin with pigmentary incontinence in the basal layer (ii) Increased melanocytes sizes with elongated dendrites *Hypopigmented macules* (i) Decrease in melanin pigments and number of melanocytes

DUH	*Hyperpigmented macules* (i) Increase in melanin with pigmentary incontinence in the basal layer *Hypopigmented macules* (i) Decreased melanin deposition in the basal layer

RAPK	*Hyperpigmented macules* (i) Increase in melanin in the basal layer with no pigmentary incontinence (ii) Hyperkeratosis with no parakeratosis

DPR	(i) Mild orthokeratosis, papillomatosis (ii) Heavily pigmented epidermis with pigmentary incontinence (iii) Interface dermatitis (iv) Superficial perivascular inflammations

NFJS	*Hyperpigmented lesions* (i) Increase in melanin in the basal layer with pigmentary incontinence

EBS-MP	*Hyperpigmented macules* (i) Epidermal atrophy (ii) Increased pigmentation in the basal cells with pigmentary incontinence

ACD	*Hyper- and hypopigmented macules* (i) Eosinophilic material (amyloid) in papillary dermis (ii) Melanin pigment incontinence

DSH: dyschromatosis symmetrica hereditaria; DUH: dyschromatosis universalis hereditaria; RAPK: reticulate acropigmentation of Kitamura; DPR: dermatopathia pigmentosa reticularis; NFJS: Naegeli-Franceschetti-Jadassohn Syndrome; EBS-MP: epidermolysis bullosa simplex with mottled pigmentation; ACD: amyloidosis cutis dyschromica.

**Table 4:** Histopathological features, electron microscopy.

Disorder	Electron Microscopy
DSH	*Hyperpigmented macules* (i) Many melanosomes in keratinocytes and few in melanocytes *Hypopigmented macules* (i) Small immature melanosomes in the melanocytes with apoptotic changes

DUH	*Hyperpigmented macules* (i) Melanosome complexes

RAPK	*Hyperpigmented macules* (i) Increased number of melanocytes in the basal layer (ii) Increased melanosomes in keratinocyte cytoplasm

DPR	*Hyperpigmented macules* (i) Increased number and size of melanosomes in the basal keratinocytes

NFJS	(i) Colloid-amyloid bodies in the papillary dermis and around sweat glands in the reticular dermis

EBS-MP	*Hyperpigmented macules* (i) Many melanosomes within the basal keratinocytes (ii) Disorganization of keratin filaments (iii) Keratinocyte vacuolization

ACD	(i) Amyloid fibrils

DSH: dyschromatosis symmetrica hereditaria; DUH: dyschromatosis universalis hereditaria; RAPK: reticulate acropigmentation of Kitamura;DPR: dermatopathia pigmentosa reticularis; NFJS: Naegeli-Franceschetti-Jadassohn Syndrome; EBS-MP: epidermolysis bullosa simplex with mottled pigmentation; ACD: amyloidosis cutis dyschromica.
